# Supplementary material for: Zyxin regulates endothelial von Willebrand factor secretion by reorganizing actin filaments around exocytic granules
Source: Nat Commun. 2017 Mar 3;8:14639. doi: 10.1038/ncomms14639 (PMC5338022; doi:10.1038/ncomms14639)
Supplement: Supplementary Information — Supplementary Figures. [file ncomms14639-s1.pdf]

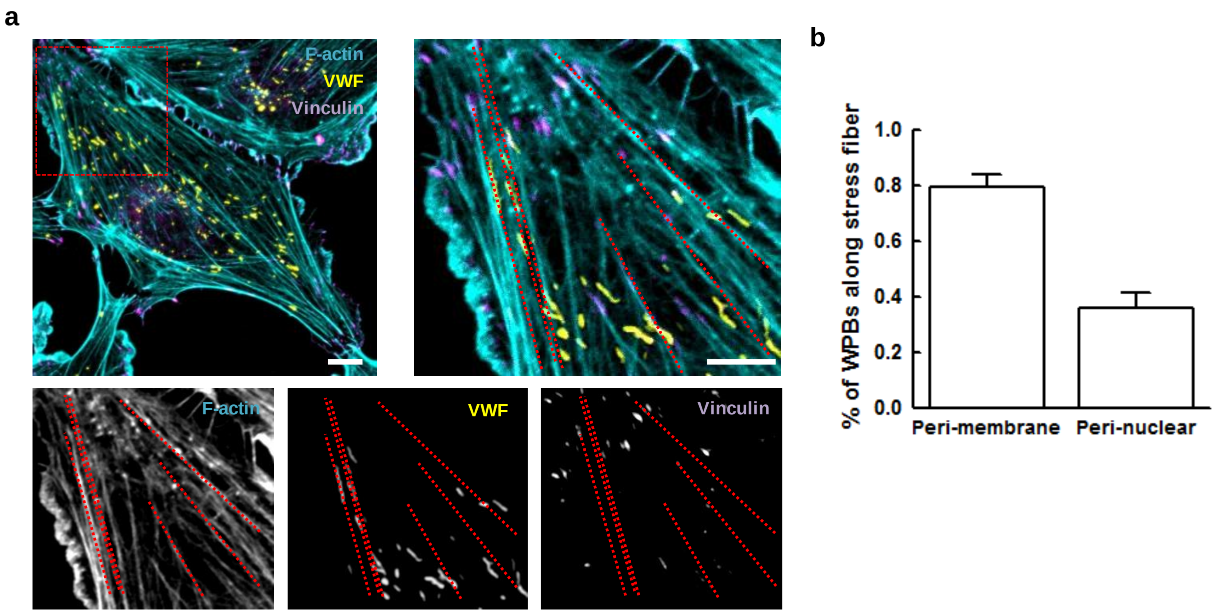

Supplementary Fig. 1. WPBs in HUVECs are located along stress fibers and at focal adhesions. (a) Immunofluorescence images of f-actin (cyan), VWF (yellow), and vinculin (magenta) by confocal microscope. The magnified insets on the right (merged) and below are the separated mono-color images, which show WPBs are located along with actin stress fibers (as indicated by red dash lines) connected to focal adhesions. (**b**) Statistic graph shows the percentage of WPBs along stress fiber (within 0.5 μm) in the peri-membrane and the peri-nuclear region. All error bars represent s.d.. Scale bars, 5 μm.

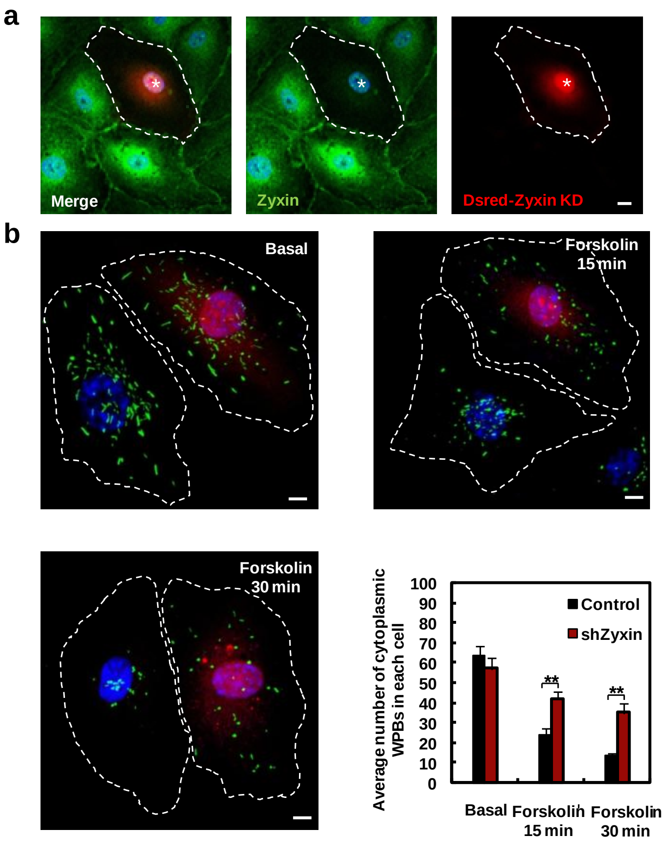

Supplementary Fig. 2. Visualization of the effect of zyxin knockdown on WPB release of HUVECs in response to forskolin stimulation. HUVECs were infected with Dsred-labeled retrovirus delivering shZyxin (the abbreviation of shZyxin-1, which is used thereafter). (**a**) Zyxin expression detected by immunostaining with an anti-zyxin antibody (green). Nuclei were labeled with DAPI (blue). Zyxin-knockdown cells expressed Dsred (red). (**b**) WPBs stained with an anti-VWF antibody (green) and nuclei co-stained with DAPI (blue). Zyxin-knockdown cells expressed Dsred (red). The images were acquired under a confocal microscope. The dash lines indicate the outline of the cells. Scale bars, 5 μm. The bar graph shows the average number of cytoplasmic WPBs per cell (n = 16, $^{**}P < 0.01$, 4 independent experiments). All error bars represent s.d.. Student's t-test was used for statistical analysis.

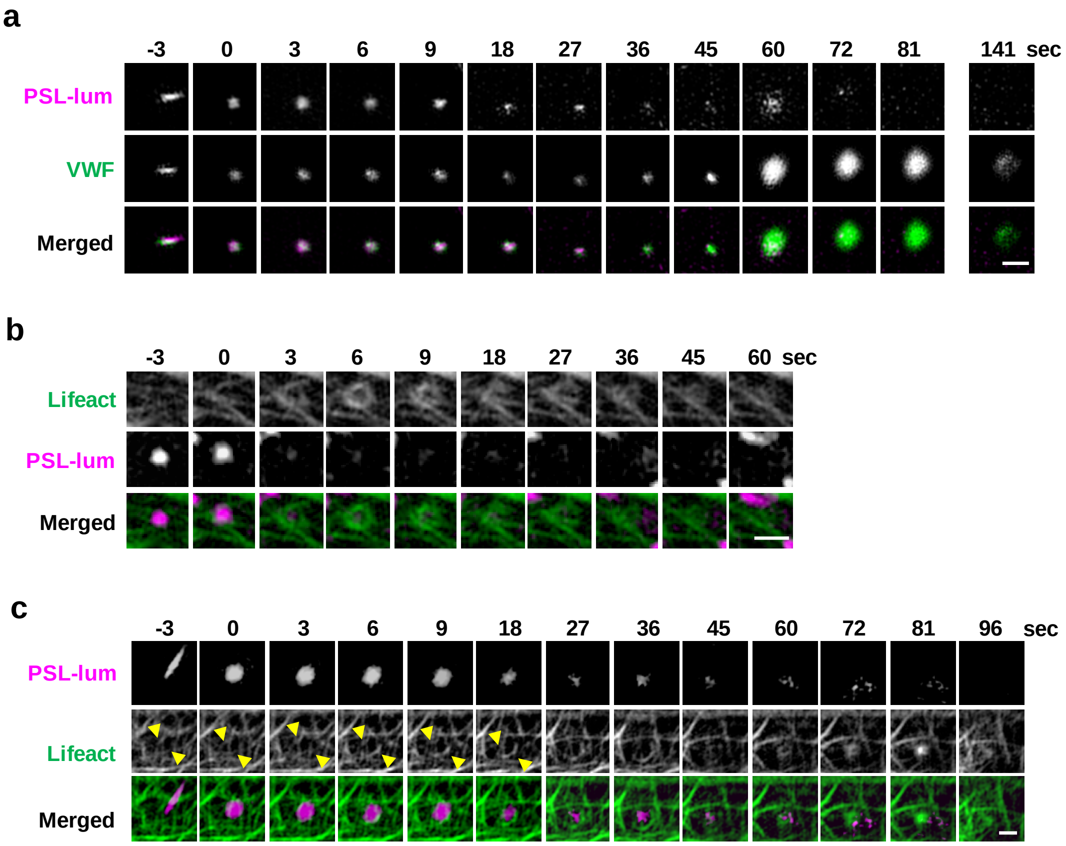

Supplementary Fig. 3. Visualization of WPB exocytosis and actin framework formation. (**a**) A HUVEC co-expressing a fusion protein of the luminal part of P-selectin with mCherry at the C-terminus (magenta) and GFP-VWF (green) was imaged with high NA TIRF-SIM. The cells were incubated in medium with forskolin. (**b, c**) A HUVEC co-expressing mCherry-PSL-lum (magenta) and GFP-Lifeact (green) in the presence of epinephrine (b) or phorbol-12-myristate-13-acetate (c) was imaged using TIRF-SIM. Images were acquired every 3 sec for 10 min. Arrowheads indicate actin framework. Time 0 is defined as fusion point. Scale bars, 500 nm.

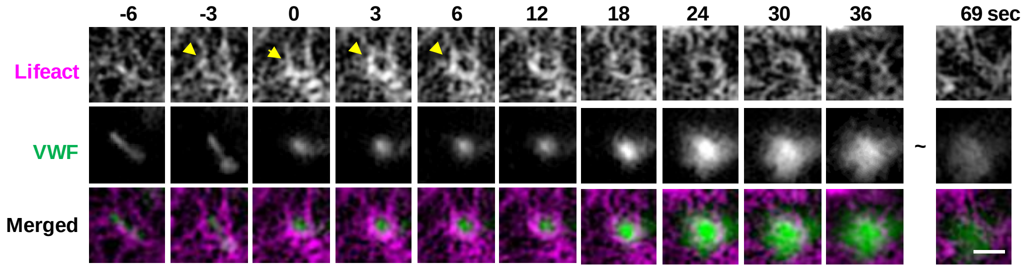

Supplementary Fig. 4. Visualization of WPB exocytosis and actin framework formation in VWF-GFP and Lifeact-mcherry expressing HUVECs. Time-lapse series showing the exocytosis of an individual WPB in a HUVEC expressing GFP-VWF (green) and mCherry-Lifeact (magenta). Arrowheads indicate actin filaments recruited to form the actin framework. Time 0 is defined as the fusion point. Scale bar, 500 nm.

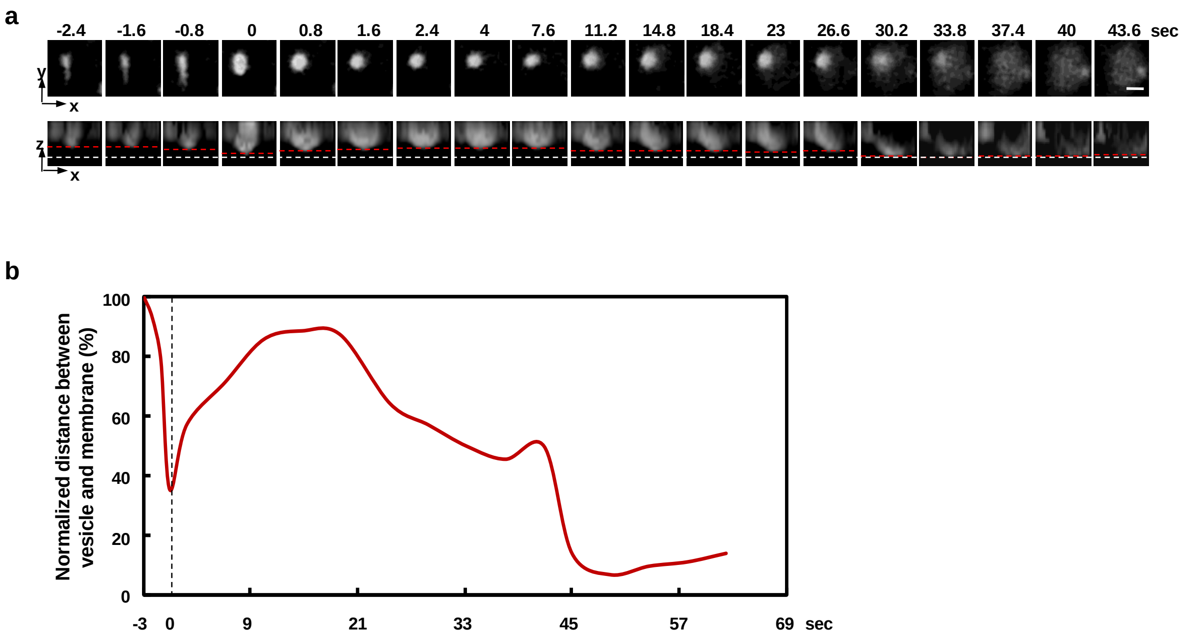

Supplementary Fig. 5. Variable-angle TIRF imaging of a WPB for 3D trajectory measurement during exocytosis. A HUVEC expressing VWF-GFP (green) was stimulated with forskolin. Images were acquired every 0.8 sec at 10 different angles for 15 min. The raw images were reconstructed using a custom program[17]. (**a**) Time-lapse series of x-y and x-z images of an individual exocytosis event. The x-z images represent the maximum intensity projections along the y axis. The red dashed line indicates the membrane-facing edge of the WPB (defined as the last pixel with a signal) and the white dashed line indicates the membrane plane (defined as the plane of clear diffusion). (**b**) The graph shows the normalized distance between the edge of the WPB plane and the membrane (defined as the ratio of their separation at each time point to that at the first time point). The dash line indicates the fusion point. The statistic analysis was performed in ImageJ. Scale bar, 500 nm.

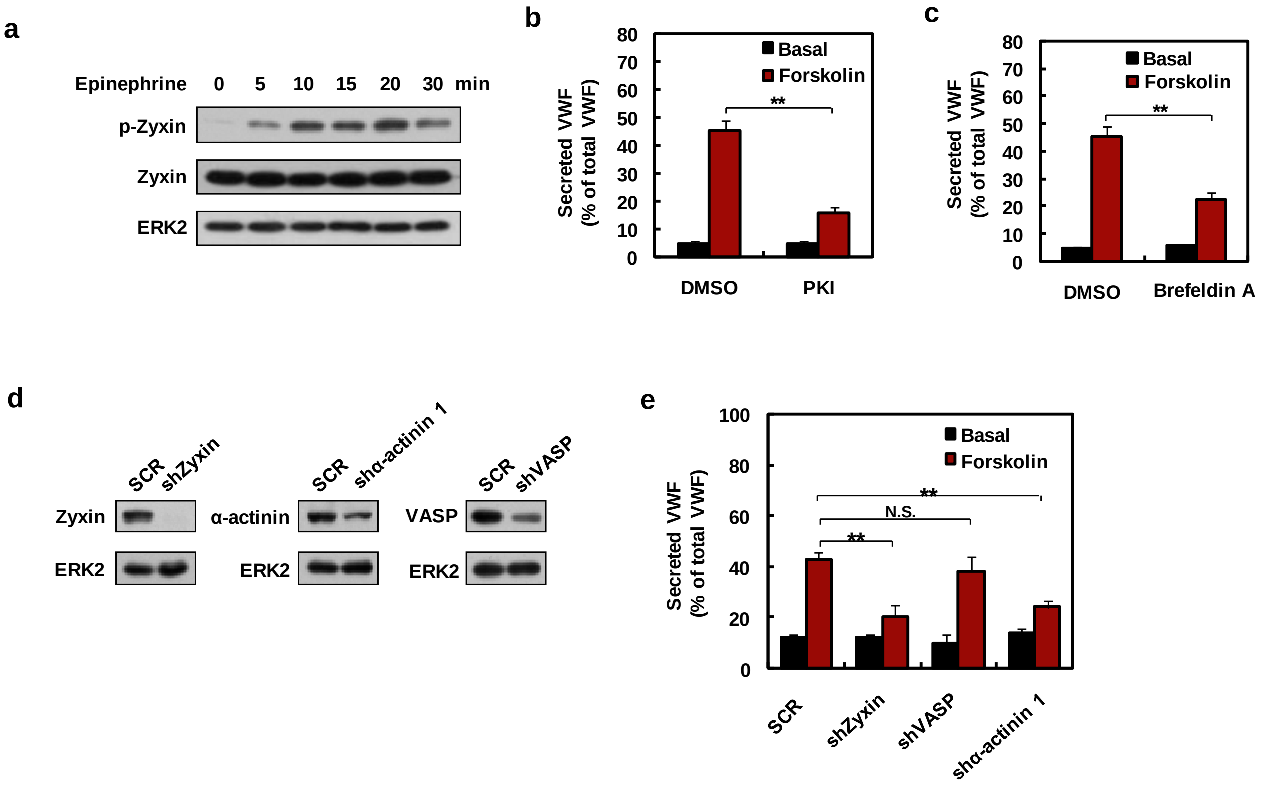

Supplementary Fig. 6. Zyxin-mediated VWF secretion depends on the PKA activity and the interaction with α-actinin. (**a**) Western-blot of phospho-zyxin (S142/143) (p-Zyxin) in HUVECs stimulated with epineprine for different time points. (**b, c**) ELISA of VWF secreted from HUVECs with forskolin stimulation in the presence of PKI (a PKA-specific inhibitor) or Brefeldin A (an EPAC-specific inhibitor). The cells were treated with (PKI) or (Brefeldin A) for 20 min or with DMSO as control, and stimulated with or without forskolin for 1 h. Bar graphs show the percentage of secreted VWF relative to cytoplasmic VWF (n = 15, $^{**}P < 0.01$ *versus* DMSO-treated cells, 4 independent experiments). (**d**) Expression levels of zyxin, α-actinin, and VASP in cells expressing scrambled or targeted shRNAs. Cells were infected with lentiviruses delivering scrambled shRNA (SCR), or shRNAs targeting zyxin (shZyxin), α-actinin 1 (shα-actinin 1), or VASP (shVASP). The cells were collected 48 h later. Western-blot analysis was used to determine the zyxin, α-actinin, and VASP expression levels. (**e**) ELISA of VWF secreted from HUVECs expressing scrambled (SCR) or targeted shRNAs (shZyxin, shα-actinin or shVASP) with forskolin stimulation (n = 12, $^{**}P < 0.01$, N.S. $P > 0.05$, 3 independent experiments). All error bars represent s.d.. Student's t-test was used for statistical analysis.

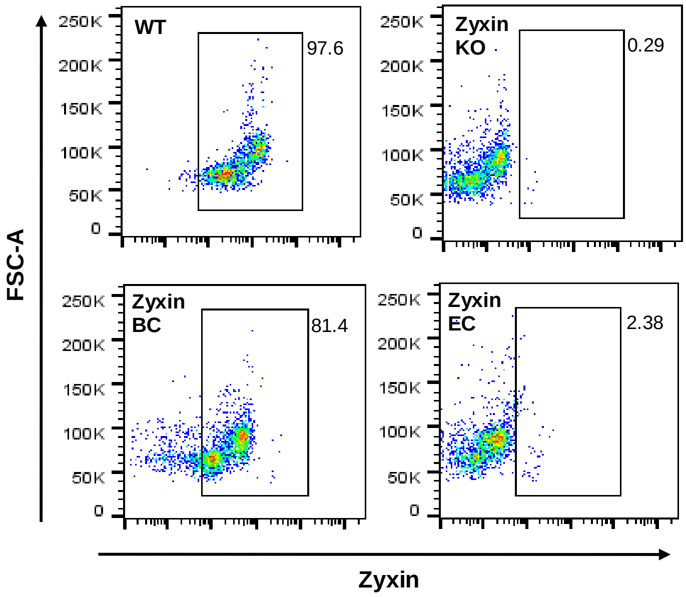

Supplementary Fig. 7. Assessment of reconstitution efficiency in cross marrow transplanted mice. Zyxin knockout bone marrow cells were transplanted in the WT recipient mice, establishing the Zyxin EC mice model (EC^{Zyx+}/BC^{Zyx-}). Meanwhile, WT bone marrow cells were transplanted into the Zyxin KO recipient to establish the Zyxin BC model (EC^{Zyx-}/BC^{Zyx+}). Representative FACS profiles of freshly isolated peripheral blood cells from WT, Zyxin KO, Zyxin BC and Zyxin EC mice at day 10 post-transplantation are shown. Cells were labeled with zyxin antibody followed by AF488-conjungated second antibody. The region indicates the class of the zyxin positive cells and the proportions were shown respectively.

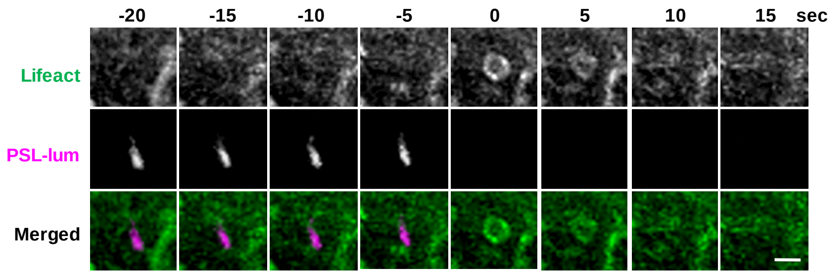

Supplementary Fig. 8. Live imaging of the dynamics of actin and WPB exocytosis with spinning disk confocal microscopy. HUVECs co-expressing mCherry-PSL-lum (magenta) and GFP-Lifeact (green) in the presence of forskolin were imaged with a spinning-disk confocal microscope. Images were acquired every 5 sec for 10 min. Z-stacks were acquired for 5 planes at steps of 500 nm. The time-lapse series shows the maximum intensity projection at each time point (n = 7, 3 independent experiments). Scale bar, 500 nm.

Fig.1 b

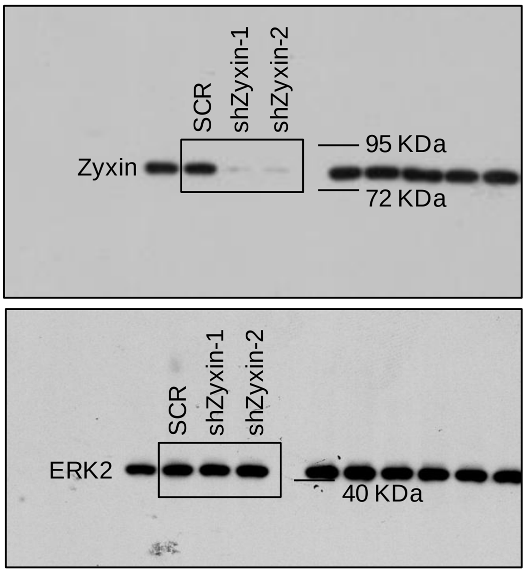

Fig. 4b

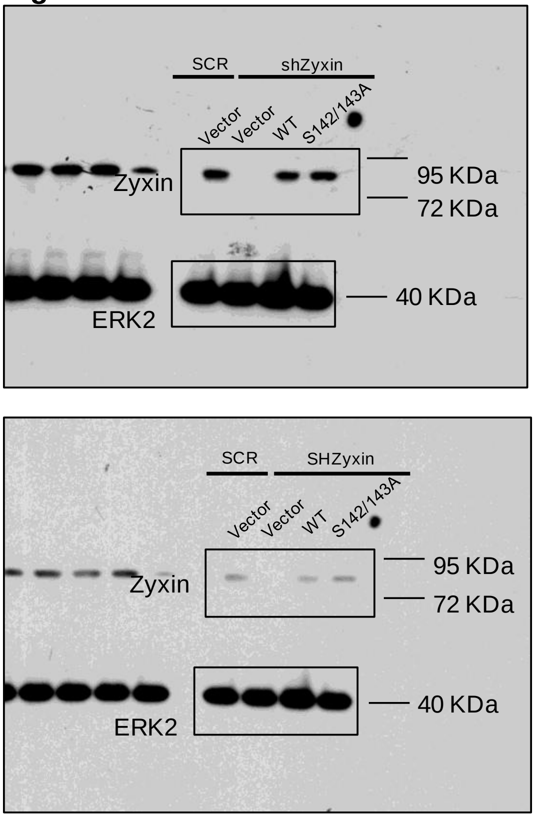

Fig.4 a

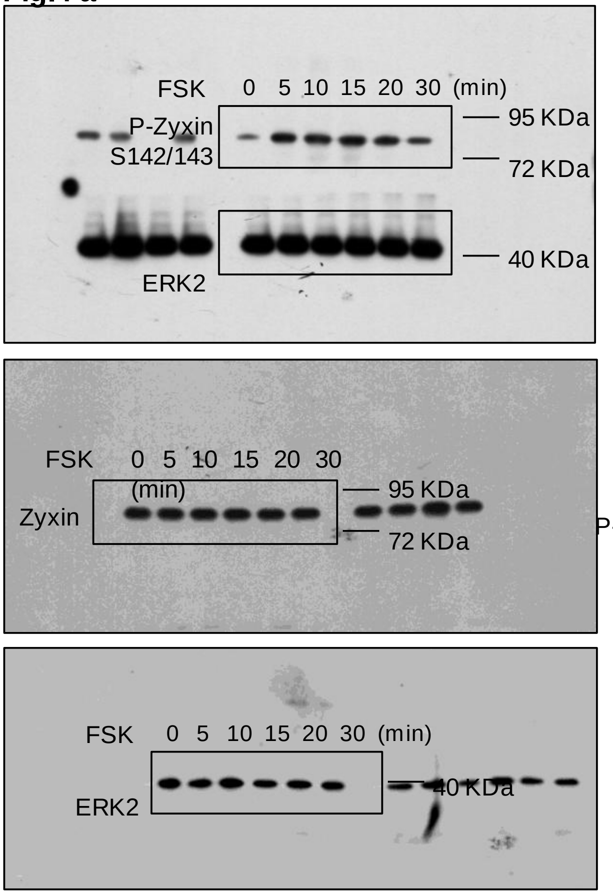

Fig. S6a

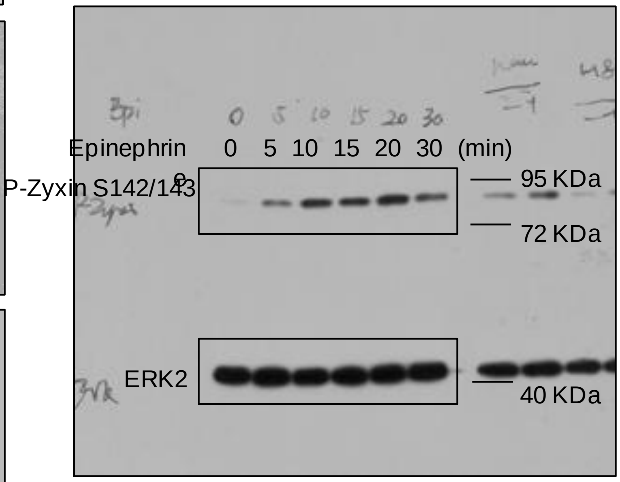

Fig. 4c left

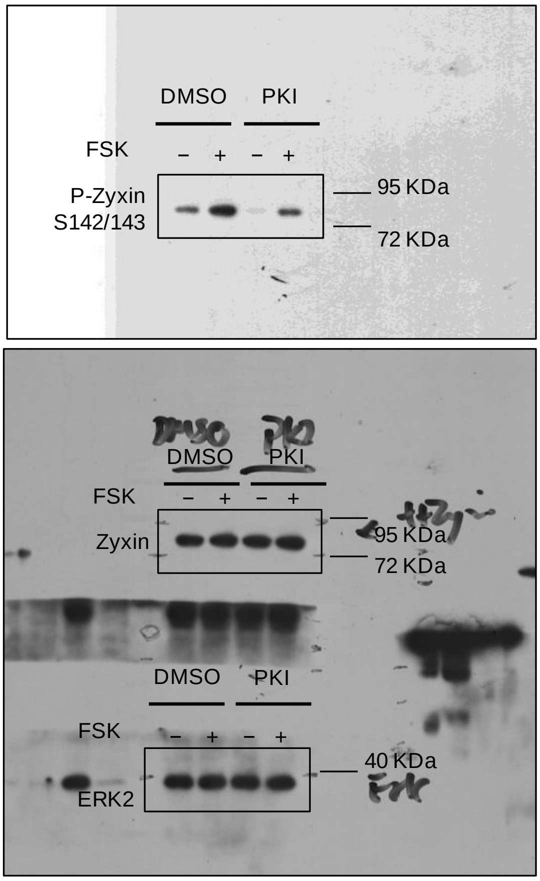

Fig. 4c right

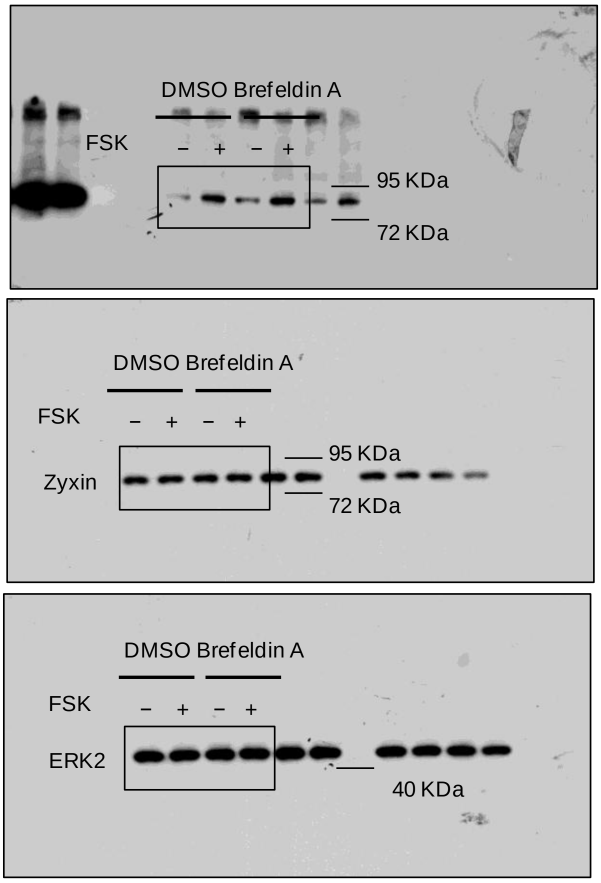

Fig. S6d

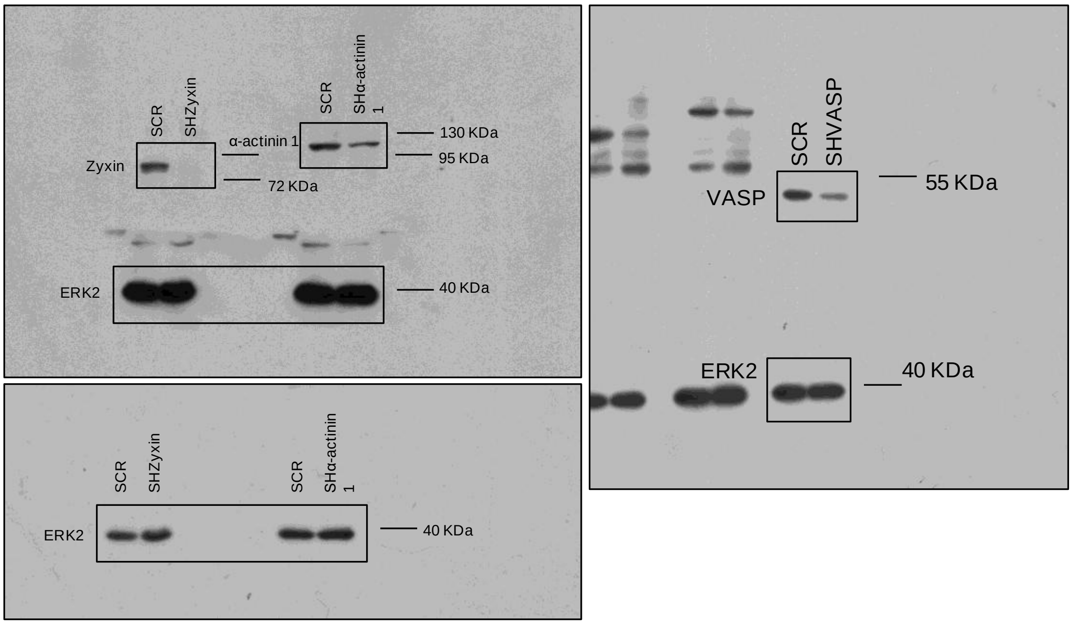

Fig. 5a

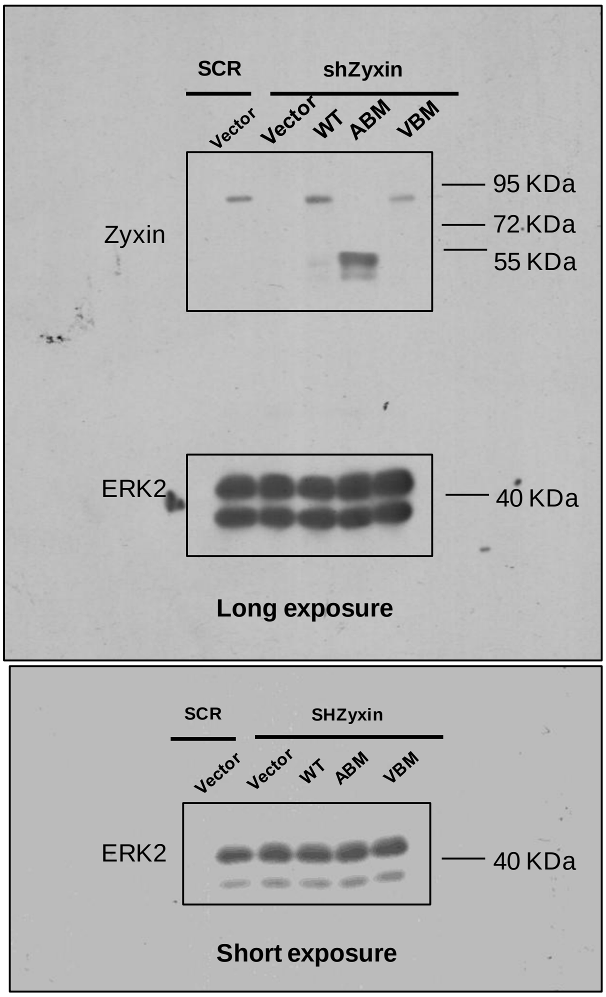

Fig. 5c

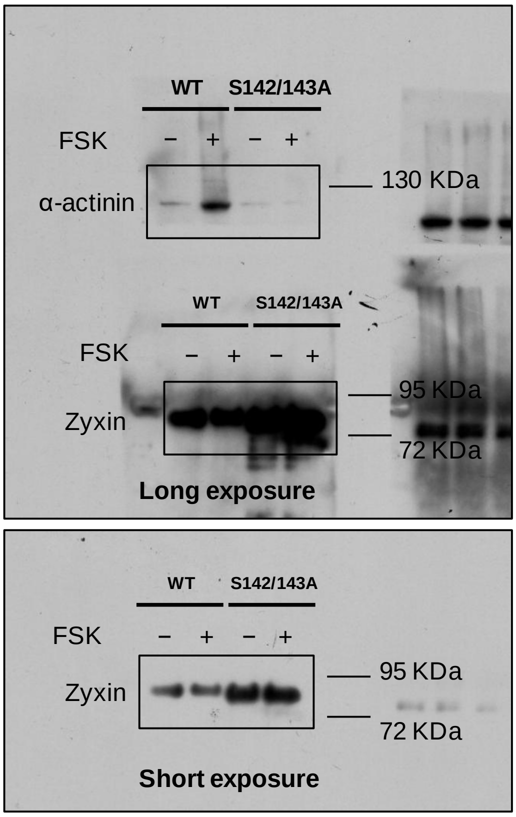

Supplementary Fig.9. The uncropped scans of western blots.
